# A high-precision multi-dimensional microspectroscopic technique for morphological and properties analysis of cancer cell

**DOI:** 10.1038/s41377-023-01153-y

**Published:** 2023-05-29

**Authors:** Lirong Qiu, Yunhao Su, Ke-Mi Xu, Han Cui, Dezhi Zheng, Yuanmin Zhu, Lin Li, Fang Li, Weiqian Zhao

**Affiliations:** 1 MIIT Key Laboratory of Complex-field Intelligent Exploration, School of Optics and Photonics, Beijing Institute of Technology, 100081 Beijing, China; 2 Department of Gastroenterology, Aerospace Central Hospital, Peking University Aerospace School of Clinical Medicine, 100081 Beijing, China; 3 Department of Pathology, Aerospace Central Hospital, Peking University Aerospace School of Clinical Medicine, 100081 Beijing, China

**Keywords:** Optical spectroscopy, Microscopy

## Abstract

Raman and Brillouin scattering are sensitive approaches to detect chemical composition and mechanical elasticity pathology of cells in cancer development and their medical treatment researches. The application is, however, suffering from the lack of ability to synchronously acquire the scattering signals following three-dimensional (3D) cell morphology with reasonable spatial resolution and signal-to-noise ratio. Herein, we propose a divided-aperture laser differential confocal 3D Geometry-Raman-Brillouin microscopic detection technology, by which reflection, Raman, and Brillouin scattering signals are simultaneously in situ collected in real time with an axial focusing accuracy up to 1 nm, in the height range of 200 μm. The divided aperture improves the anti-noise capability of the system, and the noise influence depth of Raman detection reduces by 35.4%, and the Brillouin extinction ratio increases by 22 dB. A high-precision multichannel microspectroscopic system containing these functions is developed, which is utilized to study gastric cancer tissue. As a result, a 25% reduction of collagen concentration, 42% increase of DNA substances, 17% and 9% decrease in viscosity and elasticity are finely resolved from the 3D mappings. These findings indicate that our system can be a powerful tool to study cancer development new therapies at the sub-cell level.

## Introduction

With the technological advances in nanotechnology and medical imaging over the past decades, the study of the chemical and mechanical properties of single cells and intercellular substances have become popular providing rich and comprehensive information for understanding the pathological processes of cells and humans^1–3^. Studies have shown that cancerous cells and normal cells are significantly different in terms of morphology, cytoskeleton structure, chemical properties and mechanical properties^4–6^. Malignant cancer cells have lower Young’s modulus and looser connections to the intercellular substance, which may be related to changes in the structure and concentration of certain proteins^7–9^. The mechanical and chemical properties of cancer cells and intercellular substance interact with each other to advance tumor development^10,11^.

At present, the detection methods (techniques) for mechanical properties of single cell and intercellular substance include atomic force microscopy (AFM), microfluidic technology, magnetic torsion cytometer (MTC), Brillouin spectroscopy, and other technologies^12–14^. However, except for Brillouin spectroscopy, these techniques have the limitation of not being able to measure non-contact, and will introduce stress to affect the accuracy of the measurement results. Brillouin spectroscopy is a non-destructive, non-contact and label-free detection technology of the micro-region mechanical properties of material, including spontaneous Brillouin scattering, stimulated Brillouin scattering (SBS), and surface enhance Brillouin scattering (SEBS)^15–17^. Among them, SBS system has poor stability, and SEBS technology has higher requirements on samples.

Detection methods for the chemical properties of cells and their intercellular substance include immunohistochemistry, fluorescence molecular imaging, Fourier transform infrared (FTIR) imaging, and Raman spectroscopy^18–20^. The first two of these require preprocessing such as staining, which may introduce additional interference to the research process, and FTIR cannot measure samples with high water content. Raman spectroscopy is a spectroscopic technique that detects the chemical property of substances, including spontaneous Raman scattering, stimulated Raman scattering (SRS), and surface enhance Raman scattering (SERS), etc.^21–23^. SRS could not obtain full band spectral information, and SERS had higher requirement on samples.

Combined with confocal microscopy, confocal Brillouin microscopy(CBM) enables imaging of mechanical properties of tissues and even subcellular structures^24^. Confocal Raman microscopy (CRM) has been widely used in the diagnosis, staging and treatment of cancer due to its high sensitivity and high-resolution chemical imaging capabilites^25–27^. Raman spectroscopy and Brillouin spectroscopy are different in the detection frequency range. The detection range of Brillouin spectroscopy is usually within 100 GHz, while the detection range of Raman spectroscopy is usually greater than 1 THz. However, the two can be excited and collected in a common optical path, so as to provide complementary information of mechanical and chemical properties of samples simultaneously^28^. Palombo et al. evaluated the mechanical and chemical characteristics of Barrett’s esophagus tissue sections by Raman–Brillouin spectroscopy and revealed a link between mucin water content and tissue viscoelasticity^29^. Mattana et al. characterized the viscoelastic characteristics and chemical composition of living cells by confocal Raman–Brillouin spectroscopy, and found that cell elasticity was closely related to the concentration of proteins. After oncogene expression, cell viscoelasticity occurred a significant reduction^30^.

However, due to the lack of high-precision real-time axial focusing capability of the current confocal spectral imaging technology, the size of the focusing spot will be changed due to the fluctuation of the sample in the scanning process, affecting the realization of its optimal spatial resolution. Secondly, due to the weak Raman and Brillouin scattering light and long measurement time, the traditional confocal spectral imaging system is highly susceptible to environmental interference, which leads to focus movement and ultimately affects the imaging quality. In addition, when imaging biological tissue slice samples, the optical signal of defocus layer is easily aliased with the Raman spectral signal of focus layer, and reduce the signal-to-noise ratio of the Raman spectral signal. Moreover, the relatively strong elastic background light can easily drown the Brillouin signal, and seriously reduce the extinction ratio and measurement accuracy of the system.

To solve the above-mentioned problems, we propose a method of divided-aperture differential confocal 3D Geometry-Raman-Brillouin microscopy (DDCGRBM), which combines divided-aperture laser differential confocal microscopy with Raman spectroscopy and Brillouin spectroscopy, which can achieve simultaneously in situ high-precision topography, Raman and Brillouin mapping of samples under homogenous excitation. Using the reflected light of the sample to construct a divided-aperture differential confocal system, the axial focusing accuracy could reach up to 1 nm, which ensures the stability of the system in the process of spectral detection, and keeps the system working with the optimal lateral resolution better than 400 nm. The setting of divided-aperture can effectively suppress the interference of the stray light of the defocus layer and specular reflections. We used the designed DDCGRBM to study cells and intercellular substance in gastric cancer tissue section and adjacent normal tissue section sample, and achieved high-resolution imaging of geometry, various chemical components, and viscoelasticity. The relationship between the viscoelasticity of cells and intercellular substance in tumor tissue and adjacent normal tissue and the content, structure and distribution of biological macromolecules such as protein, lipid and DNA were also discussed.

## Results

### Divided-aperture differential confocal 3D Geometry–Raman–Brillouin microscopy

**Fig. 1:**
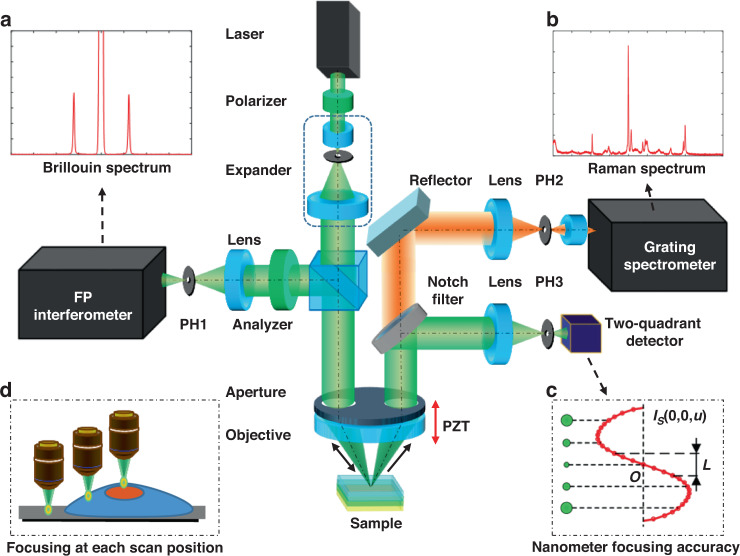
Schematic structure of the DDCGRBM system. **a** Brillouin spectrum curve at focus. **b** Raman spectrum curve at focus. **c** The differential confocal axial light intensity curve. The characteristic that the zero-crossing point of the curve is the focal position with nanometer-focusing accuracy. **d** Utilizing the ultra-high focusing accuracy of the system, high stability and optimal resolution of spectral acquisition are guaranteed at each scanning point

A schematic of the DDCGRBM system is shown in Fig. 1. The laser is collimated and expanded by a beam expander, then passes through the left side of the divided-aperture placed in front of the objective and illuminates the sample through the objective. The Brillouin scattered light is collected by the objective and passes through the left side of the divided-aperture, and is collected by the path on the same side as the illumination light path to obtain the Brillouin spectrum of the measured position of the sample (Fig. 1a). The reflected light and Raman scattered light are collected by the objective and pass through the right side of the divided aperture, and then separated by a notch filter (NF). The reflected light is reflected by the NF and then enters the divided-aperture laser differential confocal system, thereby achieving high-precision geometric imaging and focus tracking. The Raman scattered light enters the Raman spectroscopy detection system through the NF (Fig. 1b). According to our previous study, the differential confocal system is characterized by high spatial resolution and focus tracking for 3D imaging^31^.

Since reflected light, Raman scattered light, and Brillouin scattered light are all excited by the same source, DDCGRBM can synchronously provide in situ high-precision and multi-dimensional information. Due to the introduction of the divided-aperture, the optical signals between different layers are spatially separated, which can effectively reduce the interference of the noise signal of the defocused layer on the Raman spectrum at the focal point. In addition, the Brillouin scattered light and the reflected light are spatially separated, which effectively suppresses the interference of the reflected light and improves the system extinction ratio^32^ (see Supporting Information for the extinction ratio test).

In the divided-aperture differential confocal system, the zero-point *O* of differential confocal axial characteristic response *I*_s_ accurately corresponds to the focus of the DDCGRBM (Fig. 1c), thereby achieving high-precision axial focusing to ensure that the focal point of each scanning position is focused on the sample surface, and the spot size is the smallest at this time, which can achieve the optimal spatial resolution of the system (Fig. 1d)^33^. We used a normal silver mirror and a single-crystal silicon wafer to test the axial focusing capability of the system. The lateral spectral resolution of the system was tested using a square sample with silicon substrate material and PMMA pattern by the knife-edge method. The axial focusing precision of this system can reach 1 nm, and the spectral lateral resolution is better than 400 nm. For a more detailed process, see Supporting Information. The axially fixed focus working distance of the system is determined by the effective working distance of the PZT, is 200 μm in our system.

### Anti-drift ability

**Fig. 2:**
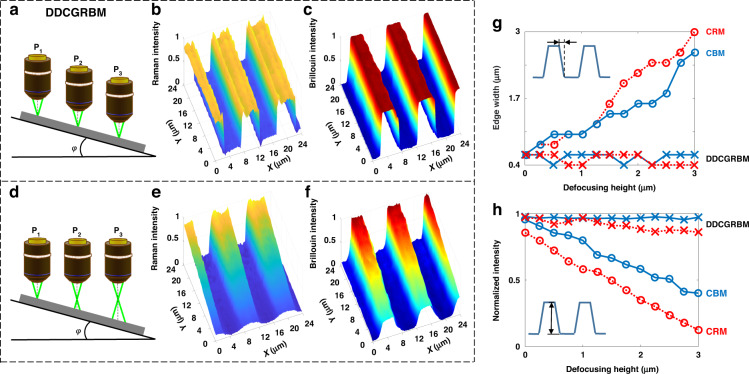
Anti-drift capability test results. The Raman spectrum exposure time is 1 s and the Brillouin spectrum acquisition time is 10 s. **a**, **d** The schematic of axial focusing capability of DDCGRBM and CRM when the sample is tilted. **b**, **e** The normalized Raman spectrum intensity map with focus tracking measured by the DDCGRBM, the normalized defocused one by CRM. **c**, **f** The normalized Brillouin spectrum intensity map with focus tracking measured by the DDCGRBM, the normalized defocused one by CRM. **g** Variation curve of step edge width with defocus height of spectral intensity maps measured by DDCGRBM, CRM and CBM. **h** Variation curve of spectral intensity with defocus height of spectral intensity maps measured by DDCGRBM, CRM, and CBM

The anti-drift ability of the DDCGRBM system was tested by using a strip-shaped polymethyl methacrylate (PMMA) sample on Si substrate. An inclination of about 7 degree was introduced to the sample to simulate a perturbated environment. The DDCGRBM system could precisely focus on at each scan position with nanometer accuracy (Fig. 2a), whereas traditional CRM and CBM systems lost focusing capability obviously (Fig. 2d). The image size was 120 × 120 pixels, and the scanning step was 200 nm. The imaging results recorded at 520 cm^−1^ Raman peak intensity mapping of the tilted sample via DDCGRBM and traditional CRM are shown in Fig. 2b, e, respectively. The imaging results recorded at 15.1 GHz Brillouin peak intensity mapping of the tilted sample via DDCGRBM and traditional CBM are shown in Fig. 2c, f, respectively.

As can be seen from Fig. 2e, f, since traditional CRM and CBM cannot track focus every time a spectrum is collected, the spectral intensity image in the upper focused area is clearer and the spectral intensity is stronger. In the lower area, which is defocused due to the tilt of the sample, the excitation spot on the sample becomes larger, resulting in a blurred image and lower spectral intensity. However, the DDCGRBM system corrects the axial position of the objective lens timely to ensure that the focal point is at the position with the smallest light spot, thus eliminating the instability caused by the change of the sample height, getting clear images in all areas (Fig. 2b, c).

As the defocusing makes the scanning spot of traditional CRM and CBM larger, the image resolution will decrease, which induced a wider edge width of the stripe pattern (Fig. 2g). At the same time, the spectral signal excitation and collection efficiency will decrease, resulting in lower spectral intensity (Fig. 2h). The DDCGRBM system has the real-time high-precision focusing capability and can ensure the focused light spot on the surface of the sample with the smallest size, which maintains the optimal resolution, spectral excitation and collection efficiency. Therefore, neither the spectral intensity nor the edge width of the spectrogram of the strip-shaped sample measured by DDCGRBM is affected by the defocusing (Fig. 2g, h).

### Ability to suppress defocused stray light interference

**Fig. 3:**
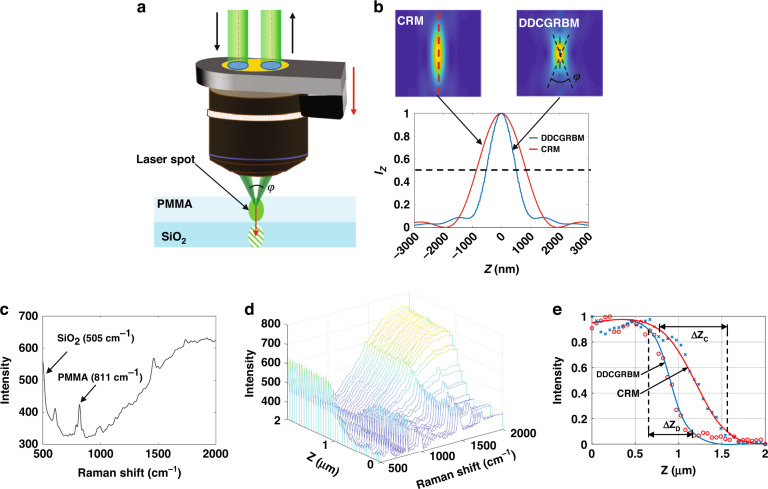
Test of suppressing defocused stray light interference ability. **a** Schematic diagram of the DDCGRBM system suppressing defocus-layer stray light interference experiment. PZT drives the objective lens to make the light spot enter the SiO_2_ from the PMMA across the interface. **b** The intensity PSF diagram of DDCGRBM and CRM in the *X* and *Z* directions when the NA = 0.9 objective is divided into the excitation optical path pupil and the collection optical path pupil with equal diameters 1/2 of the diameter of the rear pupil of the objective lens, and the *I*z plot at *X* = 0. **c** The Raman spectrum curve when the spot is located between PMMA and SiO_2_. **d** The Raman spectral curves of each scanning position during the axial scanning of the DDCGRBM system. **e** The Raman intensity changes of PMMA (811 cm^−1^) measured by the DDCGRBM and CRM systems

Compared with the traditional confocal Raman system (CRM), the DDCGRBM spatially separates the excitation light path and the collection light path of the Raman spectrum, as shown in Fig. 3a. As shown in Fig. 3b, since the PSF of the excitation light path and the collection light path at the sample form an angle *φ* in space, the size of the focal region in the axial direction is compressed^34^. Therefore, the DDCGRBM system has a stronger ability to suppress the interference of stray light in the defocused layer (see Supporting Information for details).

A double-layer transparent sample with the upper layer of PMMA and the lower layer of SiO_2_ was used to test the ability of the DDCGRBM system suppressing the interference of defocused stray light. As shown in Fig. 3a, PZT drives the objective to move the focused spot from the upper layer of PMMA to the lower layer of SiO_2_ with the steps of 50 nm, collecting Raman spectrum data of 40 points in total. Figure 3c shows the Raman spectrum curve when the focus is between PMMA and SiO_2_, and the Raman peaks of PMMA and SiO_2_ are 811 cm^−^^1^ and 505 cm^−1^ separately. It can be seen from Fig. 3d that with the movement of the light spot, the Raman spectral signal of PMMA gradually weakens, and the Raman spectral signal of SiO_2_ gradually increases. The CRM system and the DDCGRBM system were used for experiments, respectively, and the normalized position-PMMA spectral intensity curve was obtained by fitting the experimental results as shown in Fig. 3e, the slope of the curve of the DDCGRBM system is larger than that of the CRM system. Moreover, the *Z*-direction depth through which the PMMA spectral signal obtained by the DDCGRBM system (Δ*Z*_D_ = 0.51 μm) decreased from 90% of the maximum intensity to 10% was reduced by about 35.4% compared with the depth obtained by the CRM (Δ*Z*_c_ = 0.79 μm), indicating that the system has a stronger ability to separate signals between adjacent layers. Therefore, the DDCGRBM system has a better ability to suppress the interference of the defocused stray light.

### Mapping and analysis of gastric cancer tissue

**Fig. 4:**
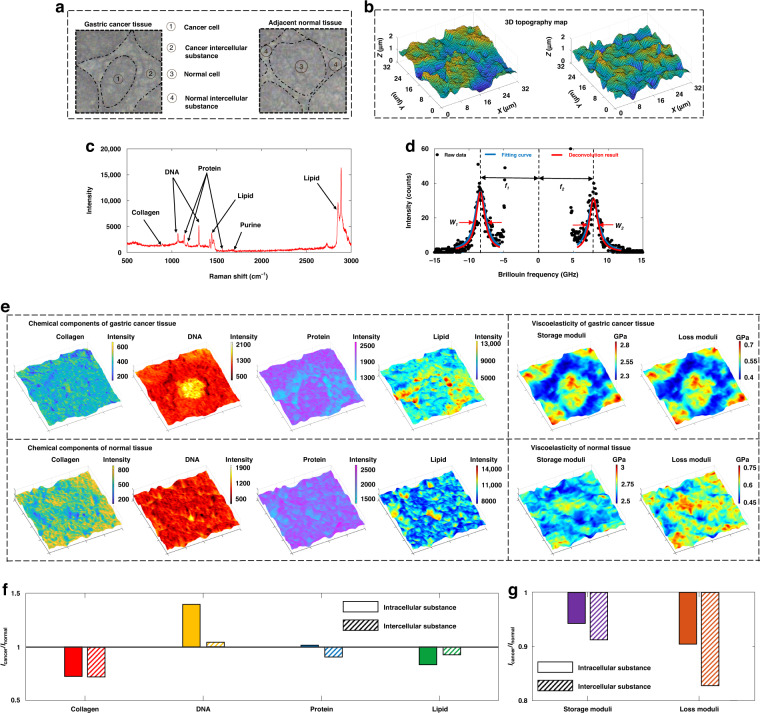
Mapping results of gastric cancer tissue and normal tissue. **a** Scanning area of gastric cancer tissue, adjacent normal tissue scanning area. Area ① is the gastric cancer cell area, area ② is the gastric cancer intercellular substance area, area ③ is the normal cell area, and area ④ is the normal intercellular substance area. **b** three-dimensional topographic map of gastric cancer tissue, adjacent normal tissue scanning area. **c** The average spectrum curve of the Raman spectrum of ten random points of gastric cancer tissue detected by the DDCGRBM system and the corresponding Raman peaks of various substances. **d** The fitting curve of the average spectrum of Brillouin spectrum of 10 random points detected by the DDCGRBM system in gastric cancer tissue. **e** The three-dimensional distribution map of various chemical components in the scanned area of gastric cancer tissue (upper left), the three-dimensional distribution map of various chemical components in the scanned area of normal tissue adjacent to cancer (lower left), The three-dimensional distribution map of loss modulus and storage modulus in the scanned area of gastric cancer tissue (upper right), and the three-dimensional distribution map of storage modulus and loss modulus in the scan area of normal tissue adjacent to cancer (lower right). **f** Concentration changes of various substances in gastric cancer cells and gastric cancer intercellular substance compared with normal cells and normal intercellular substance. **g** Changes in the storage modulus and loss modulus of gastric cancer cells and gastric cancer interstitial cells compared with normal cells and normal interstitial cells

We used the DDCGRBM system to scan and map the gastric cancer tissue and the adjacent normal tissue in the same patient (Fig. 4a). The scanning interval was 500 nm, the scanning area was 64 × 64 pixels, and the image size was 32 × 32 µm^2^. The laser intensity at the sample is about 15 mW, the integration time of Raman spectrum is 1 s, and the integration time of Brillouin spectrum is 30 s. The obtained 3D topography map can clearly reflect the cell morphology (Fig. 4b). As can be seen, the area of cancer intercellular substance is larger and the height difference between cell and intercellular substance is larger than that of normal cell. This may indicate that gastric cancer tissue has thinner intercellular substance, fewer structures such as connexins between cells, and looser connections between cells. Raman spectrum contains rich biochemical information (Fig. 4c), such as Raman peaks around 800–860 cm^−1^ can characterize collagen substances; the Raman peaks around 1080 cm^−1^ and 1330 cm^−1^ can characterize DNA substances; the Raman peaks near 1100–1300 cm^−1^ and 1550 cm^−1^ can characterize various protein macromolecules; the Raman peaks near 1450 cm^−1^ and 2840 cm^−1^ can characterize lipid substances^35,36^. The relative intensities of Raman characteristic peaks are positively correlated with the relative concentrations of characteristic substances, so using the corresponding frequency-shifted Raman peak intensities to map can characterize the distribution of corresponding substances. The original Brillouin spectrum data were deconvoluted according to the instrumental PSF to obtain the true spectral frequency shifts *f*_1_, *f*_2_ and full widths at half maximum(FWHM) *w*_1_, *w*_2_ of the Stokes peak and anti-Stokes peak (Fig. 4d). The black points are the raw data of Brillouin spectra, the blue curves are the Lorentzian fitting curves of Brillouin peaks, and the red curves are the deconvolution processed curves. The Brillouin frequency shift *f* = (*f*_*1*_ + *f*_*2*_)/2 and the FWHM *w* = (*w*_*1*_ + *w*_*2*_)/2 are related to the storage modulus and the loss modulus, which characterizes the elasticity and the viscosity, respectively^37^. The relationship between the Brillouin frequency shift *f* and the FWHM *w* and the storage modulus *M*_1_ and loss modulus *M*_2_ of the measured position of the sample is shown in Eq. (1):

1 $$\left\{ {\begin{array}{*{20}{c}} {M = M_1 + iM_2 = \rho V^2 + i\left( {\frac{{\rho V^2w}}{f}} \right)} \\ {V = \frac{{\lambda f}}{{2n}}} \end{array}} \right.$$

Where *M* is the longitudinal Brillouin modulus, *M*_1_ is the storage modulus, *M*_2_ is the loss modulus, *w* is the FWHM of the Brillouin peak, *f* is the frequency shift of the Brillouin peak, and *V* is the sound speed of the longitudinal acoustic phonons, *ρ* is the sample density, *n* is the refractive index, and *λ* is the wavelength of the incident light. Therefore, the frequency shift and FWHM of the Brillouin spectrum can be used to characterize the spatial elastic characteristics and spatial viscosity characteristics of the sample, respectively.

The three-dimensional spatial distribution map of the corresponding substances (collagen, DNA, protein, and lipid) in gastric cancer tissue (Fig. 4e, upper left) and adjacent normal tissue (Fig. 4e, lower left) can be obtained by fusing three-dimensional topographic maps and the intensity maps of different Raman spectral characteristic peaks (820 cm^−1^, 1080 cm^−1^, 1550 cm^−1^ and 2840 cm^−1^). To study the changes in chemical composition of adjacent normal and gastric cancer tissues, we calculated the average value of the corresponding Raman intensities of various substances in gastric cancer cells (Fig. 4a, area ①), gastric cancer intercellular substance (Fig. 4a, area ②), normal cells (Fig. 4a, area ③) and normal intercellular substance (Fig. 4a, area ④). The Raman intensity changes of the gastric cancer cell area/intercellular substance were compared with the normal ones, so as to obtain the concentration changes of various substances in cells and intercellular substances after cancerization (Fig. 4f). It can be seen from Fig. 4e that the distribution of collagen in gastric cancer tissue is more discrete than that in adjacent normal tissue, the collagen concentration of gastric cancer tissue is lower than that of adjacent normal tissue and 25% reduction of collagen concentration for both intracellular and intercellular substances was observed (Fig. 4f). This may be due to the sparse and loose cytoskeleton and intercellular connection structure of cancer cells, which makes cancer cells prone to shedding and metastasis. From the three-dimensional spatial distribution map of DNA substances, the distribution and concentration of DNA substances in gastric cancer cells is larger and higher than that in adjacent normal tissue and we found 42% increase of DNA substances in cancer cell area (Fig. 4f). This may be due to the abnormal proliferation behavior of cancer cells, which causes the nucleus to enlarge and the nuclear material to increase. It can be seen from Fig. 4f that there is almost no difference between the protein Raman intensity of gastric cancer cells and normal cells, but the Raman intensity in the intercellular substance of gastric cancer tissue is lower than that in normal tissue, which is related with the fact that cancer cells secrete proteases to degrade intercellular proteins, thereby enhancing their metastatic ability^38^. From the three-dimensional distribution map of lipid substances (Fig. 4e), most of the lipids in gastric cancer tissue are distributed in the intercellular substance, while the distribution in normal tissue is relatively uniform. From Fig. 4f, it can be seen that the concentration of lipid substances in gastric cancer cells and gastric cancer intercellular substance decreased significantly. This may be related to the increased nutrient consumption caused by the rapid proliferation of cancer cells and the special lipid metabolism of cancer cells^1^.

According to Eq. (1), the measured Brillouin spectral data is processed, and fused with the three-dimensional topography map to obtain the three-dimensional spatial distribution of storage modulus and loss modulus of gastric cancer tissue (Fig. 4e upper right) and adjacent normal tissue (Fig. 4e lower right), characterizing the spatial elastic characteristics and spatial viscosity characteristics of the samples. In order to study the changes in mechanical properties of normal and gastric cancer tissues, we calculated the average value of storage and loss moduli of gastric cancer cells (Fig. 4a, area ①), gastric cancer intercellular substance (Fig. 4a, area ②), normal cells (Fig. 4a, area ③), respectively. The viscoelastic changes in the gastric cancer cell area compared with the normal cell area and the viscoelastic changes in the gastric cancer intercellular substance and normal intercellular substance areas were compared (Fig. 4g). It can be seen from Fig. 4e that no matter whether gastric cancer tissue or normal tissue, the viscoelasticity of the cells in the central area and the surrounding intercellular substance are significantly different, and the viscosity and elasticity of the cells are higher than that of the intercellular substance. It can be seen from Fig. 4g that the elasticity and viscosity of gastric cancer cells are lower than those of normal cells, which may be related to the reduction of collagen concentration obtained by the Raman spectroscopy analysis as mentioned above. 17% and 9% decrease in viscosity and elasticity of intercellular substance are finely resolved as shown in Fig. 4g. The elasticity and viscosity of gastric cancer intercellular substance are also lower than those of normal intercellular substance, which may be related to the lower concentration of cancer intercellular substance protein and collagen. In addition, we also observed that the viscoelasticity of the nucleus of cancer cells was significantly higher than that of normal cells, which may be related to the increase of nuclear material caused by the abnormal proliferation of cancer cells.

## Discussion

In summary, we developed a divided-aperture differential 3D confocal Geometry-Raman-Brillouin microscopy (DDCGRBM) to simultaneously detect the multi-dimensional information, including 3D geometrical morphology, Raman spectrum, and Brillouin spectrum, which can characterize the 3D spatial distribution of topographic, chemical, and mechanical features of samples.

An axial focusing accuracy of 1 nm is achieved within the height range of 200 μm, which ensures that the system can keep the focused spot size at the smallest and the system resolution is the highest when the sample height changes. This is critical in imaging biological tissue samples. Because the size of cells is usually between several micrometers to tens of micrometers, and the size of cancer cells is larger^39^. And living biological tissue usually deforms slowly over time; high-precision focusing during scanning is essential to obtain high-resolution spectral mapping^40^.

The fluorescence of the slides can interfere with the Raman signal of cells. Moreover, the Raman spectral signals between different layers of biological tissues are also prone to crosstalk^41^. In addition, the Brillouin frequency shift of biological tissues is usually low, which is easily interfered by reflected light signals. Improving the extinction ratio is crucial for Brillouin detection of biological tissues^42^. The design of the divided-aperture can suppress the interference of defocused stray light during the detection of Raman spectroscopy, and the interference of reflected light during the detection of Brillouin spectroscopy. The noise influence depth of Raman detection reduces by 35.4%, and the Brillouin extinction ratio increases by 22 dB.

The divided-aperture differential 3D confocal Geometry-Raman-Brillouin microscopy (DDCGRBM) experiments on gastric cancer tissues and adjacent normal tissues showed that the concentration of collagen in cancer tissue is lower and the distribution of collagen is more discrete. The concentration of DNA was high and concentrated in cancer cells. The protein concentration was lower in the stroma of cancer cells. Lipid concentration is low and concentrated in the intercellular substance of cancer cells. The viscoelastic properties of cells and intercellular substance in gastric cancer tissues were lower. These confirms the previous hypothesis that denaturation and concentration changes of protein substances in cancer tissue and changes in tissue viscoelasticity lead to increased invasiveness^1^. It provides a new perspective for further understanding of cancer development process. As a novel imaging technology that can characterize the chemical and mechanical properties of cancer cells and tissues in real time and with high stability, DDCGRBM can provide the emerging cancer therapy methods based on regulating the biochemical response and viscoelasticity of cancer tissues to prevent cancer metastasis. Therefore, DDCGRBM can provide a powerful tool for research in the fields of carcinogenesis process research and cancer treatment. However, the design of divided aperture will also reduce the collection angle of the objective lens, thus reducing the collection efficiency of scattered light and resulting in the reduction of spectral signal intensity.

In this paper, we only applied DDCGRBM to the study of gastric cancer tissue section samples from a single patient. In the future, this technology can be used to obtain the similarities and differences in geometric, chemical and mechanical properties of different types of cancer by multi-information imaging of different types of cancer tissues, so as to reveal the causes of cancer, and even develop into a high-precision, label-free and contact-free cancer diagnosis tool. Cancer exhibits different physiological behaviors and biochemical reactions at different stages. DDCGRBM can be used to perform multi-information imaging of cancer tissues at different stages and study the changes in geometric, chemical, and mechanical properties of cancer cells during the development process, which can provide clues for the research of therapeutic methods. In addition, DDCGRBM also has a wide application prospect in the field of novel two-dimensional materials research due to its advantages of high-resolution simultaneous in situ multi-information imaging.

## Materials and methods

### Divided-aperture differential 3D confocal Geometry-Raman-Brillouin microscope

The DDCGRBM system was constructed according to the schematic structure shown in Fig. 1: A single longitudinal mode laser (COHERENT Verdi G2) with a wavelength of 532 nm was selected as the excitation light source. A microscope objective with a numerical aperture of 0.9, 100× (OLYMPUS MPlanFLN 100×) is driven by a piezoelectric ceramic transducer (PZT, P-725.CD, Physik Instrumente, Germany) for axial scanning imaging. A two-dimensional translation stage (P-542.2CD, Physik Instrumente, Germany) was used to move the sample for horizontal scanning imaging. Raman scattered light was collected by a high-resolution C-T grating spectrometer (FHR1000, Horiba Jobin Yvon, France). A Notch Filter (LPD01-532RU-25, Semrock, USA) was used to separate reflected light and Raman scattered light. Brillouin scattered light was collected by a tandem multi-pass F-P interferometer (JRS Scientific Instruments TFP-1). Two 10 µm pinholes (Newport, PH-10) and a two-quadrant detector (Hamamatsu Photonics S3096-02) were used to construct the divided-aperture differential confocal system.

### Sample characterization

The human gastric cancer tissue and adjacent normal tissue sections used in this paper were all provided by Beijing Aerospace Center Hospital, China. All sections were obtained by endoscopic biopsy, and were obtained after fixation, paraffin embedding and other treatments.

This work has been approved by the Ethics Committee of the Beijing Institute of Technology.

## Supplementary information

Supplementary Information for a high-precision multi-dimensional microspectroscopic technique for morphological and properties analysis of cancer cell
