# Supplementary material for: A high-precision multi-dimensional microspectroscopic technique for morphological and properties analysis of cancer cell
Source: Light Sci Appl. 2023 May 29;12:129. doi: 10.1038/s41377-023-01153-y (PMC10226997; doi:10.1038/s41377-023-01153-y)
Supplement: Supplementary file 1 — Supplementary Information for a high-precision multi-dimensional microspectroscopic technique for morphological and properties analysis of cancer cell [file 41377_2023_1153_MOESM1_ESM.docx]

** Supplementary Information for “A high-precision multi-dimensional microspectroscopic technique for morphological and properties analysis of cancer cell”**

Lirong Qiu^1^, Yunhao Su^1^, Ke-Mi Xu^1^, Han Cui^1^, Dezhi Zheng^1^, Yuanmin Zhu^2^, Lin Li^2^, Fang Li^3^, Weiqian Zhao^1^*

1. MIIT Key Laboratory of Complex-field Intelligent Exploration, School of Optics and Photonics, Beijing Institute of Technology, Beijing 100081, China
2. Department of Gastroenterology, Aerospace Central Hospital, Peking University Aerospace School of Clinical Medicine, Beijing 100081, China
3. Department of Pathology, Aerospace Central Hospital, Peking University Aerospace School of Clinical Medicine, Beijing 100081, China

Corresponding author(s)

Weiqian Zhao([zwq669@126.com](mailto:zwq669@126.com))

# 1. Axial focusing principle of DDCGRBM

As shown in Fig. S1, in the DDCGRBM system, when the sample is axially defocused, the spot position on the detection focal plane will move laterally. Therefore, placement of the point detector slightly off-axis laterally on the detection focal plane will shift the axial intensity response curve of the split pupil confocal microscopy imaging system.


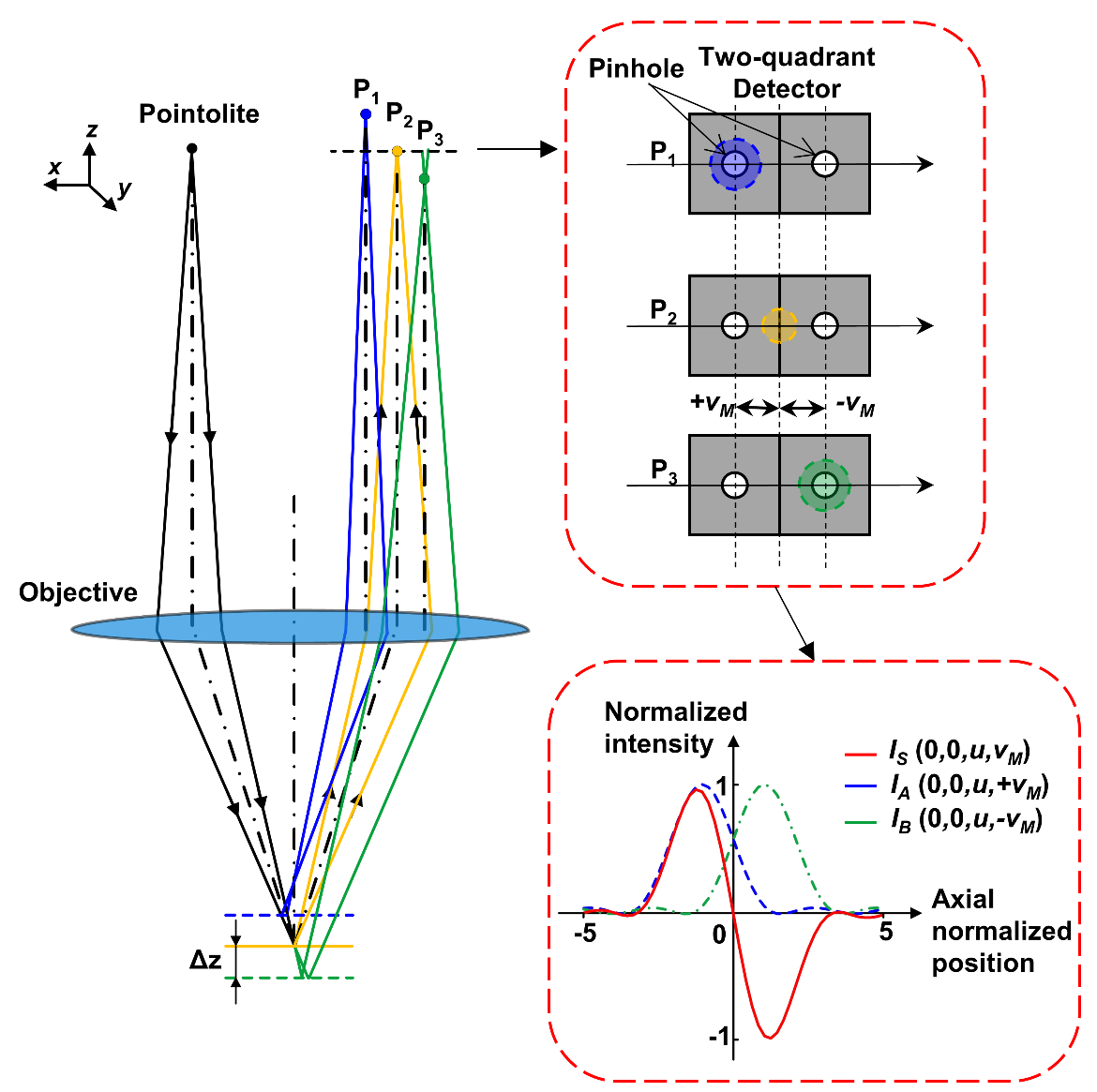


Fig. S1. Axial focusing principle of DDCGRBM

Taking advantage of this feature, two pinholes with a distance of 2*v_M_* are placed symmetrically about the confocal focal position on the x-axis of the detection focal plane of the divided-aperture confocal microscope optical path. A two-quadrant detector was placed behind the pinhole to detect the light intensity signals *I_A_*(*v_x_, v_y_,u,+v_M_*) and *I_B_*(*v_x_, v_y_,u,-v_M_*) respectively after the two pinholes. According to the coherent imaging theory[1], *I_A_*(*v_x_, v_y_,u,+v_M_*) and *I_B_*(*v_x_, v_y_,u,-v_M_*) can be expressed using off-axis point detector 3D intensity response:

 (1)

Here, *h_i_*(*v_x_*, *v_y_*, *u*) and *h_c_*(*v_x_*, *v_y_*, *u*, *v_M_*, *r_p_*, *φ*)are the illumination point spread function and the collection point spread function of the divided-aperture differential confocal microscope system, respectively:

 (2)

Here, *v_M_* is the lateral normalized offset of the detector on the detection focal plane, its size *v_M_* = 2*π*sin*α_d_M*/λ, *M* is the physical offset of the detector, *S*_1_ and *S*_2_ are the illumination pupil and the collection pupil, respectively. Subtract the detected two-way light intensity signals *I_A_*(*v_x_, v_y_,u,+v_M_*) and *I_B_*(*v_x_, v_y_,u,-v_M_*) to obtain the divided-aperture differential confocal axial response curve *I_s_*(*v_x_, v_y_,u,v_M_*). Using the characteristic that the zero point of the differential confocal curve corresponds precisely to the focal position of the system, the axial focusing capability of the system is improved, and the high-sensitivity axial focusing of the sample surface is achieved, thereby reconstructing the three-dimensional morphology of the sample.

The focal position is obtained by the divided-aperture differential confocal microscope, and the accurate axial fixation of the surface of the sample to be measured can be achieved point by point. Then, the in-situ Raman and Brillouin signals on the surface of the tested sample can be accurately detected to eliminate the influence of the sample surface height variation and system drift on the signal intensity. The system obtains the geometric topography information, Raman spectral information and Brillouin spectral information of the sample in situ through the point-by-point real-time fixed-focus method, and realizes the in-situ multispectral imaging of the sample with high spatial resolution and high stability.

# 2. DDCGRBM performance test

In order to verify the axial focusing capability of the system, a normal silver mirror and a single crystal silicon wafer were used as experimental samples. Adjust the objective to the focal point, and then drive the objective to scan at equal intervals in the axial direction through PZT, and take the minimum resolvable interval as the axial focusing accuracy of the system. Here, the axial interval of 1 nm is used to move, and each position is collected for 4 s (sampling frequency is 100 Hz). The signal obtained by the divided-aperture laser differential confocal system is shown in Fig. S2(a). As the mirror is gradually defocused from the focal point, the signal intensity is gradually weakened, and a clear step profile is formed. Therefore, the axial focusing accuracy of the system can reach 1 nm. A single crystal silicon wafer was used to test the axial focusing accuracy of the spectral detection system. Move the objective to the focal point, and the PZT drives the objective to move axially in steps of 300 nm. The spectral intensity signal obtained by the Raman spectroscopy detection system is shown in Fig. S2(b).


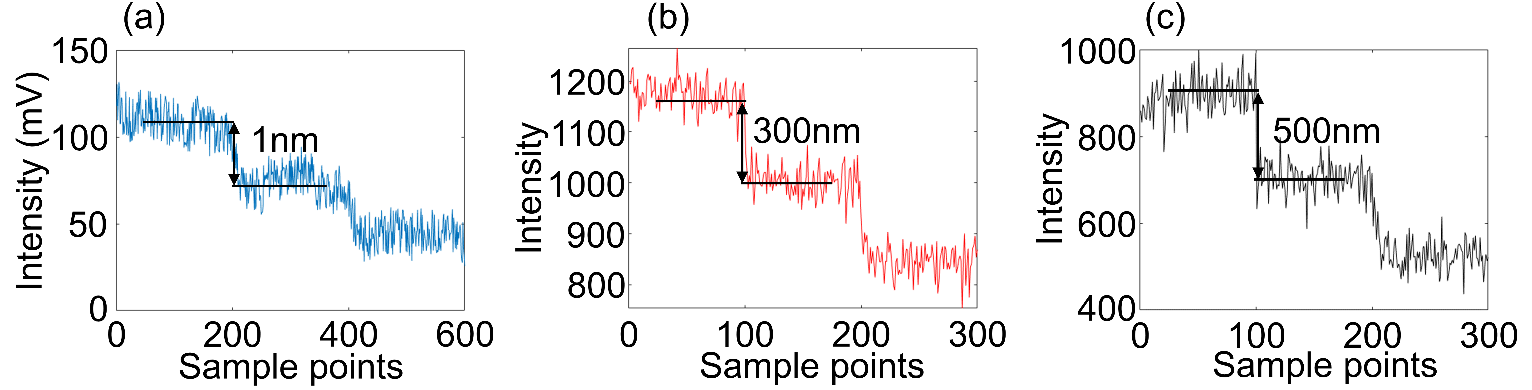


Fig. S2. Axial focusing accuracy measurement results. (a) Axial focusing accuracy of DDCGRBM. (b) Axial focusing accuracy of CRM. (c) Axial focusing accuracy of CBM.

In addition, the lateral resolution of the system was verified by using a square sample (sample height of 200 nm) with the substrate material as silicon (Si) and the sample pattern material as polymethyl methacrylate (PMMA) as the tested sample. The position-spectral intensity curves were collected in the X and Y directions across the edge of the square, respectively, as the edge spread function (ESF) in that direction[2]. The intensity of the Raman peak of Si located at 520 cm^-1^ was used as the intensity value of the Raman ESF. The intensity of the Brillouin peak of PMMA at 15.1 GHz was used as the intensity value of the Brillouin ESF. During the acquisition process, the DDCGRBM uses the differential confocal curve obtained by the reflected light to track focus on the sample surface. The objective lens changes its axial position with the change of the sample height to ensure that the focal point is always located on the sample surface, thereby minimizing the excitation spot. The number of acquisition points was 50, and the distance between the points was 60 nm. The first derivative of the ESF in the x and y directions is obtained respectively to obtain the linear spread function (LSF) in the same direction, and the full width at half maximum (FWHM) of the LSF is the lateral resolution in this direction. The FWHM results of multiple measurements are all less than 400 nm, so the spectral lateral resolution of DDCGRBM is better than 400 nm.


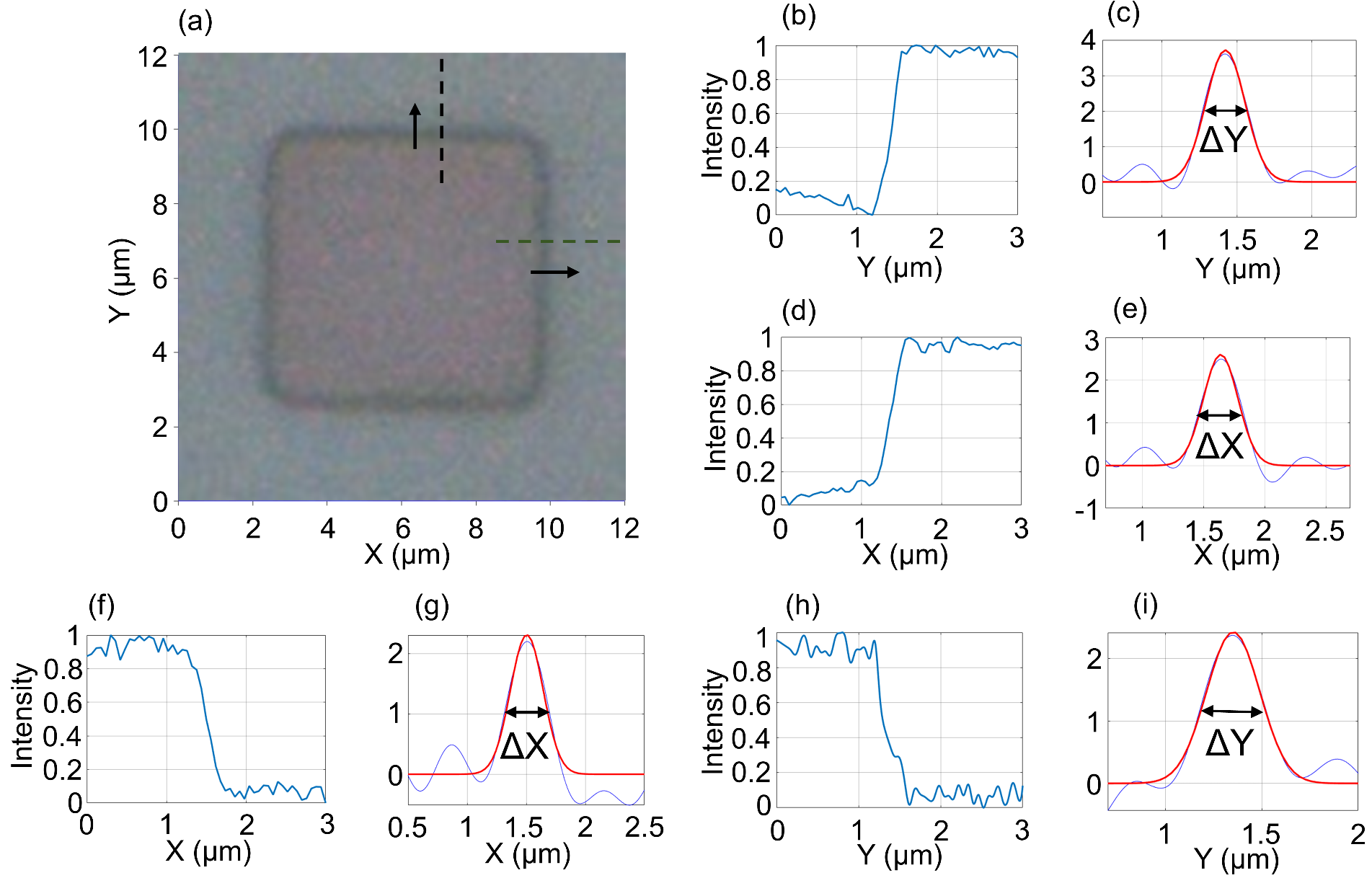


Fig. S3. DDCGRBM system lateral resolution test results. (a) Micrographs of Si-based PMMA-coated square samples. (b)、(d)、(f)、(h) are the position-spectral intensity (ESF) normalized curves of the Raman and Brillouin spectra in the Y and X directions, respectively. (c)、(e)、(g)、(i) are the first derivative curves (LSF) of the ESF curves of the Raman spectrum and the Brillouin spectrum in the Y and X directions, respectively, and the curve FWHM ΔY and ΔX serve as the lateral resolution of the system in the corresponding directions.

# 3. Principle of suppressing defocused stray light interference

The DDCGRBM system adopts the design of divided aperture to separate the illumination path and the Raman collection light path, and only intersects in the focal area of the objective lens, so as to optimize the point spread function of the system and improve the ability of the system to resist defocus signal interference. According to the incoherent imaging theory[1], for a sample with a certain depth, the spectral intensity distribution function detected by the DDCGRBM system can be expressed as:

 (3)

Here, *h_i_*(*v_x_*, *v_y_*, *u*) and *h_c_*(*v_x_*, *v_y_*, *u*, *v_M_*, *r_p_*, *φ*) are the illumination point spread function and the collection point spread function of the divided-aperture differential confocal microscope system, respectively.


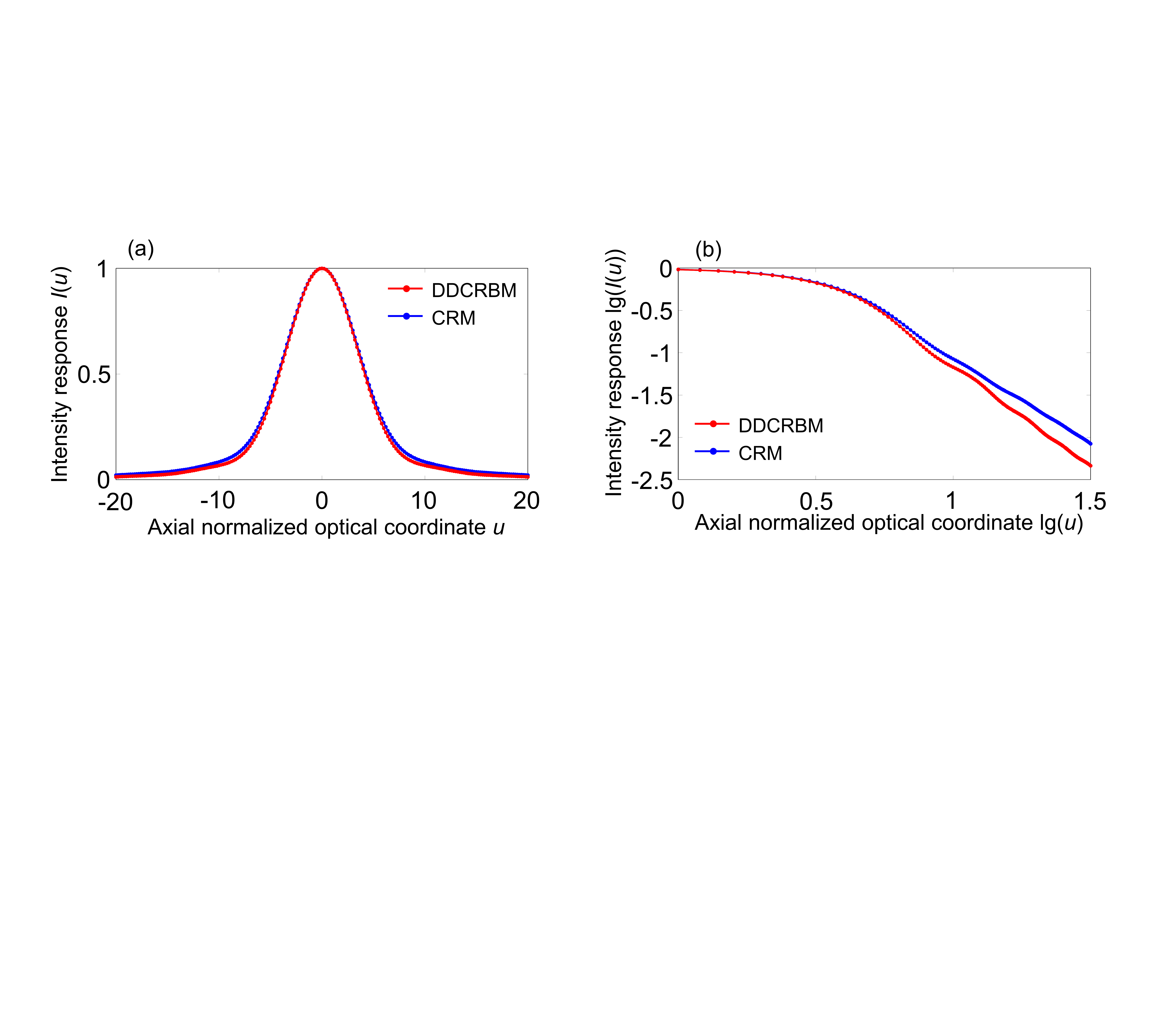


Fig. S4. Axial response curves of DDCGRBM and CRM systems. (a) uniform coordinates. (b) logarithmic coordinates.

As can be seen from the above figure, compared with the CRM system, the DDCGRBM system reduces the FWHM of the axial response curve by 2.8%, but the full width at full maximum is reduced by 23.3%. This shows that for the DDCGRBM system, the stray light signal in the defocused area has less influence on the spectral signal in the focal area, and the ability to suppress defocused stray light is significantly improved.

# 4. Brillouin extinction ratio test of DDCGRBM

The contrast of the Brillouin spectral detection system can be quantitatively described by the extinction ratio, which is defined as:

$C=10\times\lg(\frac{I_{\max}}{I_{\min}})$ (4)

Here, *I*_max_ and *I*_min_ are the maximum light intensity and the minimum light intensity detectable by the spectral detection system, respectively. The higher the extinction ratio of the spectral detection system, the larger the spectral intensity response range, so it is more suitable for weak signal detection. Only when the ratio of the non-frequency-shifted component (the background light) to the frequency-shifted component (the Brillouin scattered light) in the spectrum is lower than the extinction ratio of the system, can the Brillouin scattered light be completely detected.

In order to quantitatively compare the extinction ratio of the DDCGRBM system and the CBM system, we used a silver-coated mirror as the measured sample. Adjust the focus of the objective lens to the mirror surface, set the laser power to 1 mW, set the spectral scanning range to ±30 GHz, and set the single spectrum acquisition time to 10 s. The laser is focused on the mirror through the attenuation plate and the measuring objective lens, and generates reflected light and scattered light at the same time. The F-P interferometer is used to record the spectral intensity at different spectral frequencies. In DDCGRBM, the F-P interferometer only collects the Brillouin scattered light separated from the reflected light, while in CBM, the F-P interferometer collects both the reflected light and the Brillouin scattered light. Divide the spectral intensity at the corresponding spectral frequency detected by the F-P interferometer by the corresponding laser power and multiply it by the corresponding attenuation. The spectral intensity under each attenuation degree is scaled according to the attenuation ratio, and the test result of the extinction ratio as shown in Fig. S5 can be obtained.


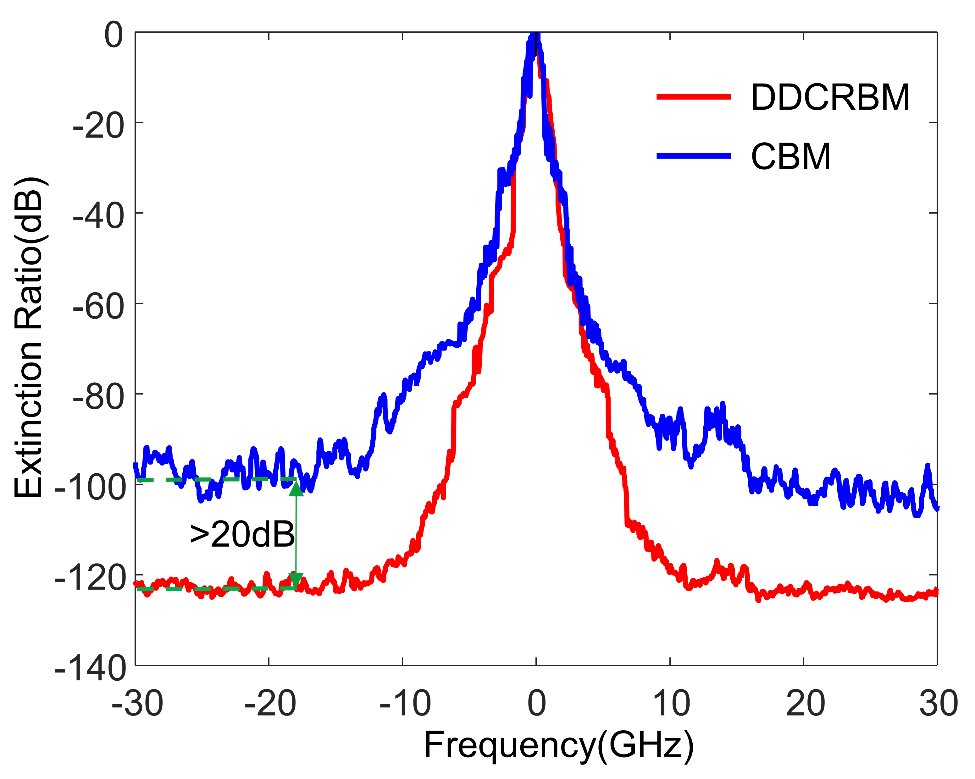


Fig. S5. Extinction ratio test results of DDCGRBM and CBM systems

It can be seen from Fig. S5 that the extinction ratio of CBM is about 100 dB, and the extinction ratio of DDCGRBM after eliminating the interference of reflected light is about 122 dB. Therefore, compared with traditional CBM, the extinction ratio of DDCGRBM is improved by about 22 dB.

# 5. Comparative analysis of imaging results between DDCGRBM system and confocal spectral microscope

The DDCGRBM system and the CRM system were used to perform Raman spectroscopy mapping experiments on the area of the gastric cancer tissue. 10 random Raman spectral curves of the two systems were selected to average and normalized for comparison. As shown in Fig. S6(a), since CRM will be interfered by the fluorescence of the slide, DDCGRBM has a stronger ability to suppress the interference of defocused stray light, the baseline of Raman spectrum of gastric cancer tissue obtained by DDCGRBM system after 1000 cm^-1^ was lower than the baseline of Raman spectrum obtained by CRM system. Therefore, the DDCGRBM system can reduce the interference of the slide fluorescence signal on the Raman signal of gastric cancer tissue. It can be seen from Fig. S6(c) that the edge of the nucleus in the 1080 cm^-1^ Raman intensity map obtained by the DDCGRBM system is clearer. It can be seen from Fig. S6(d) that the 1550 cm^-1^ Raman intensity map obtained by the DDCGRBM system can show more detailed spatial distribution of intracellular proteins. This is because the DDCGRBM system fixes the focus in real time during the point-by-point scanning spectrum acquisition process, ensuring that the sample is located at the position with the smallest excitation spot each time the Raman spectrum is collected, which ensures the realization of the optimal spatial resolution, so the information in the obtained Raman intensity map is more abundant.


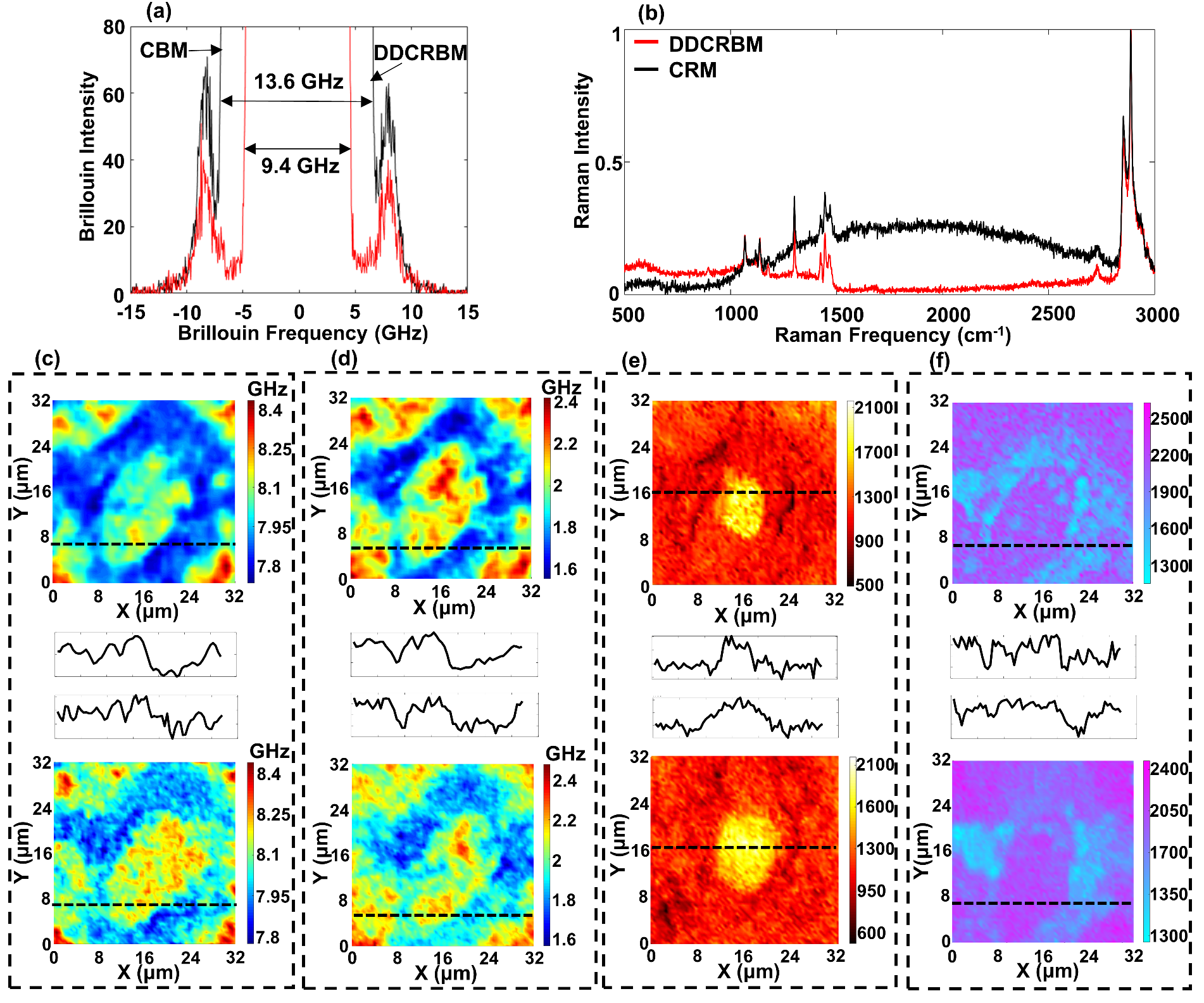


Fig. S6 (a) Raman curve of gastric cancer tissue obtained by DCRBM system and CRM system. (b) Brillouin curve of gastric cancer tissue obtained by DDCGRBM system and CRM system. (c) Raman intensity map of gastric cancer tissue obtained by DDCGRBM and CRM system at 1080 cm^-1^. (d) Raman intensity map of gastric cancer tissue obtained by DDCGRBM and CRM system at 1550 cm^-1^. (e) Brillouin frequency shift map of gastric cancer tissue obtained by DDCGRBM and CRM systems. (f) Brillouin FWHM map of gastric cancer tissue obtained by DDCGRBM and CRM systems.

Brillouin spectroscopic mapping experiments were performed on the region of gastric cancer tissue using the DDCGRBM system and the traditional CBM system, respectively. 10 random Brillouin curves of the two systems were selected and averaged for comparison. As shown in Fig. 6S(b), since the divided-aperture system spatially separates the reflected light from the collected Brillouin scattered light, the extinction ratio of the system is improved [3], the reflected light signal width at 0 GHz is reduced from 13.6 GHz to 9.4 GHz. Since the viscoelastic information is characterized by the frequency shift and FWHM of the Brillouin spectrum, it is necessary to fit the measured Brillouin spectrum to extract the frequency shift and FWHM information. However, if the reflected light signal is too strong, part of the Brillouin signal will be overwhelmed, resulting in distortion of the fitting result. Comparing the Brillouin frequency shift map (Fig. S6(e)) and the FWHM map (Fig. S6(f)) obtained by the DDCGRBM system and the CBM system, it can be seen that due to the interference of reflected light, the CBM system has large frequency shift and half-width fitting errors, resulting in a low image signal-to-noise ratio. However, the DDCGRBM system has a high extinction ratio due to point-by-point focusing scanning, so the measured Brillouin spectrum frequency shift map and half-width map are smoother and the outline of cancer cells is clear.

# 6. Divided aperture structure

The divided aperture used in this system is aluminum physical divided aperture placed in front of the objective lens, and its structure is shown in Figure S7. The gray area is light blocking area, the white area is light passing area, the left light passing area is the excitation and Brillouin collection aperture, and the right light passing area is the reflected light and Raman collection aperture.


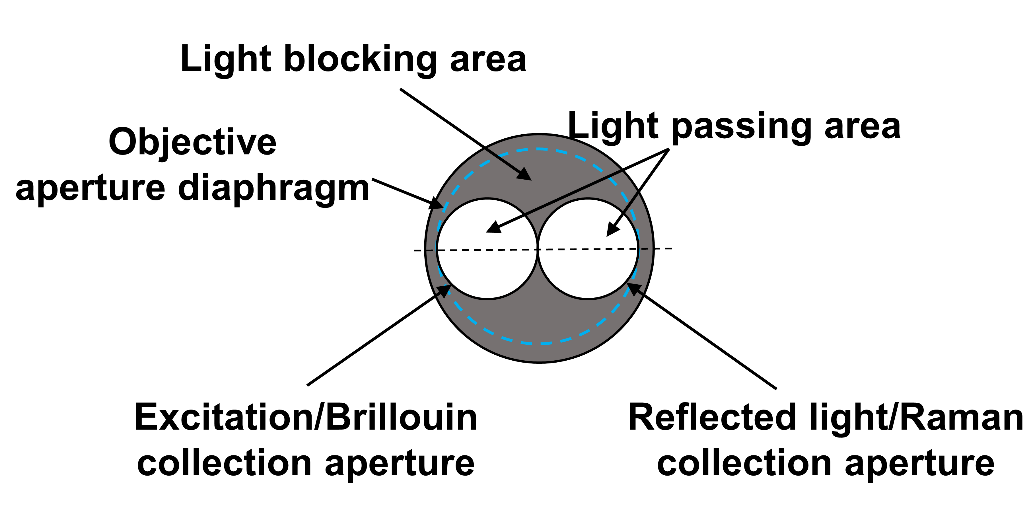


Fig S7. Schematic diagram of divided aperture structure

# 7. Fresh cancer tissue and adjacent normal tissue experiments

We used DDCGRBM to collect the Raman and Brillouin spectra of fresh cancer tissues and adjacent normal tissues that had been dried for different times, and the results are as follows. As can be seen from the fig. S8(a)-(d), the main changes in the Raman spectra during drying are that the intensity of the Raman peak of the hydroxyl group between 3100 and 3800 cm^-1^ becomes lower, and the main changes in the Brillouin spectra are that the frequency shift and FMHF become larger. These changes were the same in cancer and adjacent normal tissue samples. It can be seen that paraffin embedding does have a great effect on the viscoelastic characteristics of the samples, but the effect is the same for the cancer tissue and the normal tissue, so it does not affect the comparison of the viscoelastic differences between the intracellular and the intercellular substance.

*
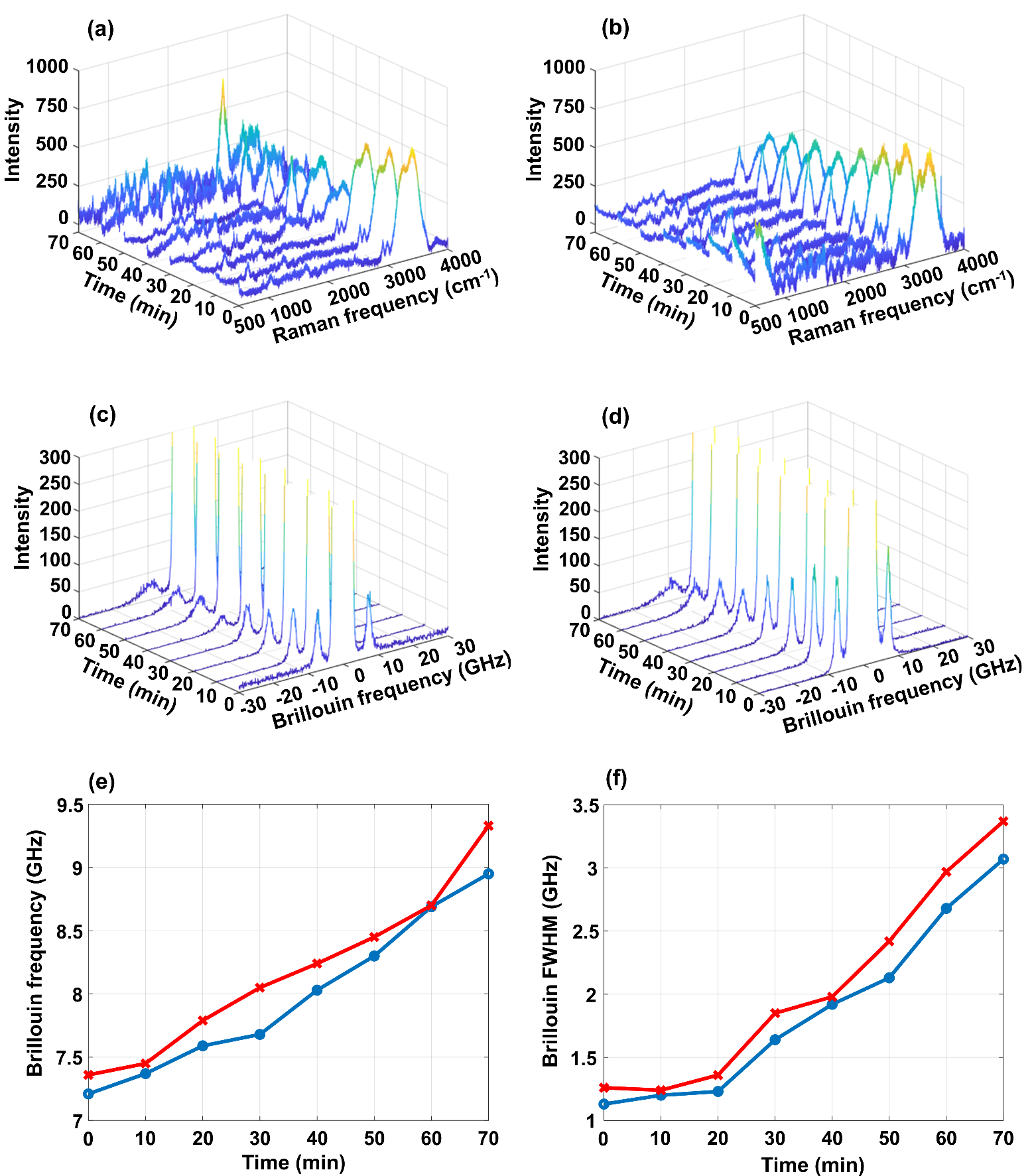
*

Fig.S8 (a) Raman spectra of adjacent normal tissue at different times. (b) Raman spectra of colon cancer tissue at different times. (c) Brillouin spectra of adjacent normal tissue at different times. (d) Brillouin spectra of colon cancer tissue at different times. (e) Brillouin frequency shifts of adjacent normal tissue and colon cancer tissue at different time points. (f) Brillouin FWHMs of adjacent normal tissue and colon cancer tissue at different time points.

# 8. Comparison of DDCGRBM and traditional methods

The traditional spectrophotometer, Kjeldahl method and Soxhlet extraction apparatus were used to compare the test results obtained by DDCGRBM, which verified the validity of the data and the correctness of the analysis conclusion.

We first used DDCGRBM system to collect Raman spectra of fresh colon cancer tissue and adjacent normal tissue, and the results were shown in Fig. S9(b). Then, the concentrations of DNA, proteins and lipids in fresh colon cancer tissues and adjacent normal tissues were measured by spectrophotometer, Kjeldahl method and Soxhlet extraction apparatus, and the results were shown in Table 1. The Raman spectra of colon cancer tissue and adjacent normal tissue were normalized with the Raman intensity of hydroxyl group (3100-3800 cm^-1^).

Table 1. Concentrations of proteins, DNA and lipids obtained by Kjeldahl method, spectrophotometer and Soxhlet extraction apparatus.

| Components  Samples | Proteins concentrations | DNA concentrations | Lipids concentrations |
| --- | --- | --- | --- |
| Colon cancer tissue | 11.13% | 103.9 μg∙mL^-1^ | 9.19% |
| Adjacent normal tissue | 12.48% | 87.96 μg∙mL^-1^ | 10.10% |
| Differences | -1.35% | 15.94 μg∙mL^-1^ | -0.91% |


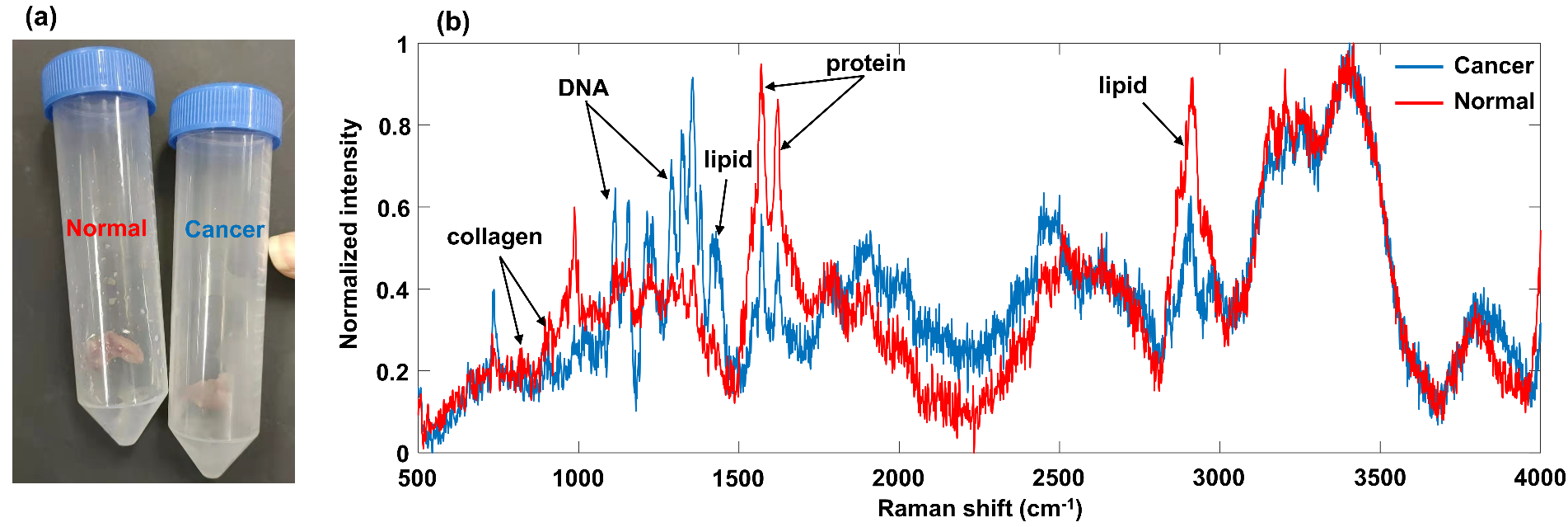


Fig. S9. (a) Colon cancer tissue and adjacent normal tissue. (b) Raman spectroscopy of colon cancer tissue and adjacent normal tissue.

It was found that the Raman intensity of collagen, protein and lipid in colon cancer tissue (820 cm^-1^, 1550 cm^-1^, 2840 cm^-1^) was significantly lower than that in normal tissue. The Raman intensity of DNA (1080 cm^-1^) was significantly higher than that of normal tissue. The concentration of DNA in colon cancer tissue measured by spectrophotometer was higher than in normal tissue. The protein concentration of colon cancer tissue obtained by Kjeldahl method was lower than that of normal tissue. The lipid concentration of colon cancer tissues obtained by Soxhlet extraction apparatus was lower than that of normal tissues. It can be seen that the biological data measured by DDCGRBM system is reliable.

# 9. morphological and imaging performance of the DDCGRBM system

We use a standard step sample (HS-500MG) calibrated by atomic force microscope (Dimension3100) as a sample to test and compare the morphological and imaging performance of the DDCGRBM system. Fig. S10 (a) shows the AFM scanning results, and Fig.S10 (b) shows the section height curve. The measured step edge width Δ*x*_A_ is about 0.25 μm, and the step height Δ*h*_A_ is about 508.8 nm. Fig. S10 (c) shows the results of DDCGRBM scanning and Fig. S10 (d) shows the curve of section height. The measured step edge width Δ*x*_D_ is about 0.4 μm, and the step height Δ*h*_D_ is about 506.7 nm.


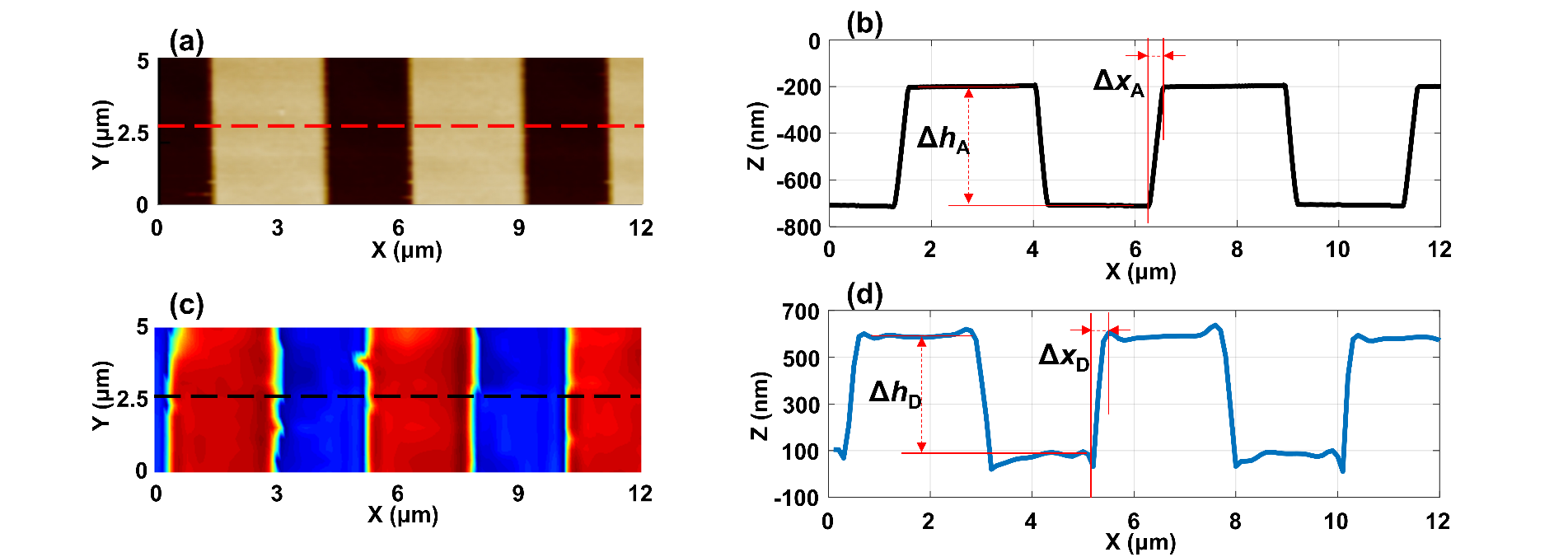


Fig. S10 (a) AFM scanning results of standard step sample. (b) Section height curve of AFM measurement results. (c) Results of DDCGRBM scanning of standard step samples. (d) Section height curve of DDCGRBM measurement results.

**References**

[1] Gu, M. Principles of Three-Dimensional Imaging in Confocal Microscopes (WORLD SCIENTIFIC, 1996).

[2] Yamazaki T. et al. A method to measure the presampling MTF using a novel edge test device and algorithm. Proceedings of SPIE - The International Society for Optical Engineering. 5368:696-704 (2004).

[3] Wu, H. et al. Divided-aperture confocal Brillouin microscopy for simultaneous high-precision topographic and mechanical mapping. Optics Express. 28(21):31821 (2020).
